# Development of the Longitudinal Study of Health and Ageing in Kenya (LOSHAK)

**DOI:** 10.1093/geroni/igad111

**Published:** 2023-09-30

**Authors:** Niranjani Nagarajan, Shane D Burns, Roselyter M Riang’a, Eunice Muthoni Mwangi, Shaheen Sayed, Muthoni Gichu, Kenneth M Langa, Edward Miguel, Anthony K Ngugi, Joshua R Ehrlich

**Affiliations:** Department of Ophthalmology and Visual Sciences, University of Michigan, Ann Arbor, Michigan, USA; Leonard Davis School of Gerontology, University of Southern California, Los Angeles, California, USA; Department of Population Health, Aga Khan University, Nairobi, Kenya; Department of Population Health, Aga Khan University, Nairobi, Kenya; Department of Pathology, Aga Khan University, Nairobi, Kenya; Division of Geriatric Medicine, Ministry of Health, Nairobi, Kenya; Institute for Social Research, University of Michigan, Ann Arbor, Michigan, USA; Department of Internal Medicine, University of Michigan, Ann Arbor, Michigan, USA; Department of Economics, University of California, Berkeley, California, USA; Center for Effective Global Action, University of California, Berkeley, California, USA; Department of Population Health, Aga Khan University, Nairobi, Kenya; Department of Ophthalmology and Visual Sciences, University of Michigan, Ann Arbor, Michigan, USA; Institute for Social Research, University of Michigan, Ann Arbor, Michigan, USA

**Keywords:** Air pollution, Dementia, Health and Retirement Study, Healthy aging, Sub-Saharan Africa

## Abstract

In Kenya, the number of adults aged ≥60 is expected to nearly quadruple by 2050, making it one of the most rapidly aging countries in Sub-Saharan Africa (SSA). Accordingly, we designed the Longitudinal Study of Health and Ageing in Kenya (LOSHAK) to generate novel data to address the health and economic consequences of this demographic transition. Specifically, LOSHAK will investigate the social, economic, environmental, biological, and policy processes that shape late-life health and economic well-being in Kenya. Modeled on the U.S. Health and Retirement Study (HRS), LOSHAK joins a network of harmonized studies on aging in >45 countries worldwide; however, LOSHAK will be only the 2nd such study in SSA. The current feasibility and pilot phase of LOSHAK will validate measures and data collection procedures in a purposive sample of Kenyan adults aged ≥45 years. We have linguistically and culturally translated instruments while aiming to maintain harmonization with both existing HRS network studies and the ongoing Kenya Life Panel Survey. The current phase of LOSHAK is nested within the Kaloleni/Rabai Community Health and Demographic Surveillance System on the coast of Kenya. LOSHAK will advance population aging research in low- and middle-income countries through the study of (a) *biomarkers and physiological measures*; (b) *the impacts of air pollution and climate vulnerability*; (c) *Alzheimer’s disease and related dementias, mental health, disability, caregiving, and psychosocial wellbeing*; and (d) *economic security, including the impact of social welfare*. LOSHAK will inform future public health and economic policy to address challenges related to rapid aging in Kenya and throughout SSA. Accordingly, this paper aims to introduce and provide a description of LOSHAK and its aims and objectives, as well as to inform the scientific community of current study activities being used to build toward the full population-representative study.

**Translational Significance:** Populations in Sub-Saharan Africa (SSA), including Kenya, are projected to age rapidly over the coming decades. Yet, there is scarce population-level data from this part of the world to address economic and health-related challenges associated with rapid demographic and epidemiological transitions. The Longitudinal Study of Health and Ageing in Kenya (LOSHAK) has been designed to address this gap. It will provide nationally representative data on key determinants of health and economic well-being among older Kenyan adults. Data from LOSHAK will enable evidence-based public policy, public health, and economic responses to the challenges associated with population aging in SSA.

The United Nations (UN) estimates that the world population will grow to 10.8 billion in the year 2100, of which 35% (3.78 billion) will reside in Sub-Saharan Africa (SSA; Economic & Social Affairs, 2022). Projections show that in SSA, 2.37 billion adults will be over the age of 60 by 2100, accounting for 63% of the region’s population (Vollset et al., 2020). Kenya is expected to be at the heart of this rapid demographic transition, with its population aged 60 and older projected to more than quadruple over three decades, from 2.57 million in 2020 to 10.7 million in 2050 (He et al., 2020). Population aging is strongly associated with increased morbidity and an increase in the prevalence and severity of disability (McCracken & Phillips, 2017). Like other low- and middle-income countries (LMICs), Kenya is experiencing a “double burden of disease”; although the primary causes of morbidity and mortality remain infectious, maternal/neonatal, and nutritional, noncommunicable diseases (NCDs) like Alzheimer’s Disease and Related Dementias (ADRD), cardiovascular and respiratory diseases, and depression have increased in prevalence and will continue to do so (Gyasi & Phillips, 2020).

More than three-quarters (79.5%) of older Kenyans live in rural areas (He et al., 2020), where environmental exposures, climate vulnerability, availability of healthcare resources, and social ties differ from urban and peri-urban settings. Although many live in large multigenerational households (Zimmer & Dayton, 2005), a significant and growing number live alone in isolated homesteads (Fakoya et al., 2020; Mapoma & Masaiti, 2012), predisposing them to an increased risk of loneliness, social isolation, ADRD, and mental health conditions (Barnes et al., 2004; Fratiglioni et al., 2004). Historically, national health priorities in Kenya have been largely centered around the health needs of children and working-aged adults, with less emphasis on older adults and prevalent late-life conditions like dementia and sensory impairment (Guerchet et al., 2017). In fact, there are scant data on the prevalence of ADRD in Kenya and surrounding countries (Farina et al., 2020). For example, although the Global Burden of Disease Project estimates very large increases in the prevalence of ADRD in East Africa (357%) by 2050, they acknowledge that these projections are grounded in demographic changes and have less basis in current prevalence and incidence data from the region (Nichols et al., 2022). A recent systematic mapping review of aging research in SSA noted the lack of aging research from that region outside of South Africa (Kalu et al., 2021). A sign of the growing need to address health and well-being in this segment of the population, the Kenyan Ministry of Health’s Health Policy plan for 2030 identifies the importance of research among older people to help facilitate evidence-based interventions (Mauti et al., 2022).

Aging populations around the world are also particularly vulnerable to the causes (e.g., air pollution) and effects (e.g., heat waves, drought) of climate change. Indeed, population aging is likely to amplify the impacts of air pollution and extreme temperatures on morbidity and mortality due to the high prevalence of chronic diseases, social isolation, and declining physiological protective mechanisms (Chen et al., 2020). The 2017 *Lancet* Commission report on dementia prevention, intervention, and care included air pollution as a potential modifiable risk factor for dementia (Livingston et al., 2017). A recent systematic review on air pollution and dementia found a positive association between cognitive decline and particulate matter exposure, specifically to PM2.5 (Weuve et al., 2021). Although not widely studied in LMICs, air pollution is associated with neuroinflammation and accumulation of amyloid and particulate matter in the brain, thus increasing the risk of ADRD (Iaccarino et al., 2021). Moreover, prolonged heat waves and droughts, key impacts of climate change in Kenya, may increase the risk of mortality and mental health conditions (Cianconi et al., 2020).

Few studies to date on SSA have investigated risk factors for late-life disease, disability, and economic distress at the population level. The Longitudinal Study of Health and Ageing in Kenya (LOSHAK; loshak.org) will address this critical data gap. The study is part of a growing global network of panel studies on aging that are harmonized with the U.S. Health and Retirement Study (HRS; Juster & Suzman, 1995), as well as from several LMICs like CHARLS in China, CRELES in Costa Rica, MARS in Malaysia, MHAS in Mexico, LASI in India, and HAALSI in South Africa, among others. Thus, in addition to providing vital data on health, economics, and aging in Kenya, LOSHAK will also expand opportunities for researchers to make cross-national comparisons of risk factors and biomarkers across a diverse group of LMICs.

Prior to LOSHAK, the only African study in the HRS network was Health and Aging in Africa: A Longitudinal Study of an INDEPTH Community in South Africa (HAALSI), which is a longitudinal population-based study of about 5,000 adults aged ≥40 years in rural Agincourt, South Africa (Gomez-Olive et al., 2018). The foci of HAALSI include cardiovascular health, HIV, cognitive function, and dementia in a predominantly rural population. Another population-based study, the Study of global AGEing and adult health (SAGE), was not harmonized with HRS but drew small nationally representative samples in Ghana and South Africa (Phaswana-Mafuya et al., 2012). Both of these studies derived samples from the Health and Demographic Surveillance System (HDSS), a research infrastructure that collects longitudinal data on population health and demographic indicators in the region. It is a surveillance system that continuously monitors and tracks health indicators in selected communities or populations (Kaneko et al., 2012). The LOSHAK investigators plan for Wave 1 of LOSHAK to be nationally representative of the Kenyan population aged 45 years and older.

The Kenyan Life Panel Study (KLPS) is another study with which LOSHAK is closely collaborating to develop harmonized measures and data collection protocols. The KLPS began in 1998 as a randomized controlled trial of a deworming intervention in school children around Busia, Kenya. Through long-term follow-up, KLPS evaluates the health, educational, economic, and intergenerational impacts of this intervention with implications for public policy on poverty, health, education, and economic growth in Kenya and the wider region (Baird et al., 2008; Hamory et al., 2021; Miguel & Kremer, 2004). The fifth wave of KLPS, which began piloting in early 2023, will also collect data on cognition, psychosocial, and behavioral health, other self-reported health measures and risk factors, disability, sleep patterns, physical activity, air pollution exposure, socioeconomic and demographic measures, and social contacts and capital.

The current feasibility and pilot phase of LOSHAK is funded by the National Institute of Health/National Institute of Aging (R21AG077042) and the University of Michigan Center for Global Health Equity, with additional support from the Harmonized Cognitive Assessment Protocol Network (U24AG065182), and the Michigan Center on the Demography of Aging’s HRS Partner Studies Network (P30AG012846). LOSHAK will fill critical data gaps by creating the infrastructure to enable population-based research that is needed to inform policies and programs to optimize late-life health and economic well-being in Kenya and, more generally, in SSA.

## Study Objectives

The overarching goal of the current feasibility and pilot phase of LOSHAK is to build infrastructure and test measures and data collection protocols to launch a future LOSHAK that will likely be a nationally representative panel study of health and aging in Kenya. To facilitate this, we have the following objectives:

1. Assess the feasibility and acceptance of survey measures, biomarker collection, and air pollution monitoring protocols in a purposive sample of older adults in Kilifi, Kenya.
2. Determine the acceptability of measures and data collection protocols through examination of consent rates and data missingness.
3. Adapt and validate existing HRS network measures and data collection protocols in the Kenyan context.
4. Develop strong harmonization with KLPS, adopting the same linguistic and cultural translations and data collection protocols across the two studies.
5. Make de-identified data publicly available to researchers via online repositories (e.g. National Archive of Computerized Data on Aging).
6. Leverage experience gained and data collected in the feasibility and pilot stage to inform planning for a nationally representative Wave 1 LOSHAK.

## Methods and Analysis

### Study Design

The current stage of LOSHAK includes both feasibility and pilot testing (Thabane et al., 2010). Feasibility testing will include assessment of acceptance of measures, consent rates, interrogation of missingness, and cultural applicability, whereas pilot testing will include psychometric analyses. We will adopt a cross-sectional survey design to achieve our study objectives in the current feasibility and pilot phase. Subsequent full population-representative waves of LOSHAK will adopt a longitudinal panel survey design. The LOSHAK survey protocol consists of the following sections: self-reported cognitive abilities, cognition, psychosocial, mental health and behavioral, other self-reported health measures and risk factors, environmental exposures and impacts, economics, caregiving exposure and stress, physiological measures, and biomarker collection. The list of key topic areas and corresponding measures and domains can be found in Table 1. An emphasis during the current feasibility and pilot phase is on the testing of instruments that require significant adaptation or that may be most influenced by local cultural norms and interpretations. A summary of the aims and outcomes of the feasibility and pilot phase can be found in Table 2.

**Table 1.** LOSHAK Survey Measures and Domains

Measure type	Topic area	Measures ^a^	Domain
Survey measures	Cognition	SMSE	Orientation, memory, visuospatial, attention/speed, language/fluency
		10-word recall: immediate and delayed	Memory
		Animal naming	Retrieval fluency, language
		Logical memory	Memory
		Clock drawing	Executive function, visuospatial
		Making change	Numeric ability
	Psychosocial, mental health and behavioral	CES-D	Depressive symptoms
		3-Item loneliness scale	Loneliness
		CASP-19	Subjective well-being
		Ill treatment	Ill treatment
		Diener’s and single-item life satisfaction scale	Life satisfaction
		MacArthur ladder	Subjective social status
		4-Item perceived stress scale	Stress
		Single item financial strain	Financial strain
Other measures	Environment	Air pollution	
		Climate vulnerability	
		Food and Water insecurity	
	Economics	Household income	
		Retirement	
		Cash transfers	
		Household assets	
	Caregiving	Exposure and stress	
	Physiological/anthropometric	Blood pressure	
		Grip strength	
		Height	
		Weight	
		Waist circumference	
		Hip circumference	
	Biomarkers	Dried blood spots	
		Consent for future DNA sequencing	

*Notes*: CASP-19 = Control, Autonomy, Self-Realization, and Pleasure (quality of life scale); CES-D = Center for Epidemiological Studies-Depression Scale; LOSHAK = Longitudinal Study of Health and Ageing in Kenya; SMSE = Swahili Mental State Examination (adapted from the Hindi Mental State Examination [Ganguli et al., 1995]).

^a^ The final list of measures and its corresponding tools will be finalized following results from the feasibility and pilot phase.

**Table 2.** Feasibility and Pilot Phase Aims and Outcomes

Aim	Outcomes
Acceptance of measures	Translations, pre-testing of survey instrument
Consent rates	Calculation of consent rates for each measure
Interrogation of missingness	Analysis of patterns of “don’t know,” “refused,” “illiterate” responses and missing data
Cultural adaptation	Common interpretation of survey items in cognitive interviewing and pretesting
Psychometric analysis	Internal consistency, correlations, confirmatory factor analysis model fit indices
Publish data	Make publicly available through online data repositories
Plan Wave 1 LOSHAK	Use experience and data from current phase to plan a nationally representative panel study

*Note*: LOSHAK = Longitudinal Study of Health and Ageing in Kenya.

### Study Tools

The cognitive module consists of a battery of neuropsychological and cognitive tests that are commonly employed in other HRS partner studies (Langa et al., 2020) and have been harmonized with the KLPS cognitive module that has undergone extensive pre-testing in Kenya. Cognitive tests include the 21-item Swahili Mental State Examination (SMSE; an adaptation of the Hindi Mental State Examination; Ganguli et al., 1995) that has been employed and validated in the Longitudinal Aging Study in India (LASI), 10-word recall—immediate and delayed (Moms et al., 1989), animal naming (Rosser & Hodges, 1994), logical memory test (Wechsler, 2009), clock drawing (Agrell & Dehlin, 2012), and making change (a simple computational question).

The psychosocial, mental health, and behavioral module includes questions on depressive symptoms (Radloff, 1977), loneliness (Hughes et al., 2004), subjective well-being (Hyde et al., 2003), ill-treatment (International Institute for Population Sciences & California, 2020), life satisfaction (Diener et al., 1985; Lucas & Brent Donnellan, 2012), financial strain (Kahn & Pearlin, 2006), the MacArthur ladder (Adler et al., 2000), and the 4-item perceived stress scale (Cohen et al., 1983). Following this, we ask participants about their overall health status and the presence of various disabilities (e.g., sensory impairments, difficulty walking, etc.).

The subsequent section on environmental exposures asks about household fuel use, issues related to climate vulnerability, and food and water insecurity. Select participants will also be outfitted with an air pollution monitoring device (AtmoTube Pro, AtmoTech Inc., San Francisco, CA, USA) to directly measure exposure to pollutants (e.g., PM2.5). The economics section of the instrument aims to assess household income, retirement information, governmental cash transfers, and household assets. Finally, caregiving exposure and stress will be assessed to understand both the positive and negative impacts of caregiving on the participant.

After the survey instrument is complete, LOSHAK interviewers will obtain physiological and anthropometric measurements, including blood pressure, grip/hand strength, height, weight, waist, and hip circumference. In addition, participants will be asked to provide dried blood spots (DBS). In the current feasibility and pilot phase, several DBS assays will be conducted in Kenya and in replicate at a reference laboratory at the University of Washington, USA. Finally, we will ask participants whether they would be willing, in the future, to provide saliva samples for DNA sequencing. All consent forms and survey questionnaires have been translated into Swahili, the local language in Kilifi, Kenya, and underwent back translation, following WHO standards to ensure rigorous translation (Ozolins et al., 2020).

### Sampling Strategy

In the current feasibility and pilot phase of LOSHAK, we are building on the existing Kaloleni/Rabai Community Health and Demographic Surveillance System (KRHDSS) in coastal Kenya (Ngugi et al., 2020). Through biannual surveys, KRHDSS tracks the health of about 98,000 individuals, approximately 14,000 of whom are aged ≥45 years (the LOSHAK sampling frame). The KRHDSS will provide a uniquely robust platform and a well-characterized population for feasibility testing and initial pilot testing for LOSHAK.

All participants in the current feasibility and pilot phase will be recruited from the KRHDSS study sample. We will use purposive sampling to ensure approximately equal numbers by gender, rural, and peri-urban dwelling, and the following age strata: 45–55, 56–65, and >65 years (accounting for 41%, 31%, and 28% of KRHDSS population aged ≥45 years, respectively). During this phase, we will recruit approximately 200 individuals for testing of all survey, physiological, and molecular biomarker measures (approximately 30 participants for air pollution monitoring). The sample size for survey measures was determined based on data required to conduct psychometric analyses with adequate precision (Linacre, 1994).

During subsequent nationally representative waves of LOSHAK, we plan to employ a stratified area cluster design based on geographically and administratively well-defined Community Health Units, geographic designations, and villages. This will be undertaken in collaboration with the Kenya National Bureau of Statistics (KNBS). Through the National Sample Survey and Evaluation Program, KNBS develops, maintains, and regularly updates a nationally representative master sampling frame that provides a basis for the implementation of household surveys in Kenya. Ultimately, sampling decisions, as well as response rates at the household and individual levels, will be incorporated into the calculation of sampling weights to enable the estimation of population parameters.

### Recruitment and Fieldwork

A major objective of the current feasibility and pilot phase of LOSHAK is to gauge the acceptability of survey measures, anthropometric measures, biomarker collection, and air pollution monitoring among study participants from the KRHDSS cohort. Recruitment of participants will take place in two stages. In the first stage, a LOSHAK interviewer team will establish contact with the community by visiting in person and engaging in activities to build rapport with relevant stakeholders including community health volunteers, community leaders, local government administration, and health system management. When the LOSHAK team reaches the community, they establish contact with the Community Health Unit leader/Community Health Volunteers who will then introduce them to other stakeholders. The team will provide the stakeholders with all study information and answer questions. In the second stage, interviewers will contact pre-identified participants to obtain an appointment for an agreeable time for the face-to-face interview. On the day of the interview, interviewers will explain the study in detail to the participants and obtain informed consent. If the participant is unable to answer the survey, a proxy informant will be identified by the participant and consent will be obtained from the proxy. All consent procedures have been approved by the Aga Khan University (AKU) Institutional and Scientific and Ethics Review Committee (approval number 2022/ISERC—109 (v3)).

If an identified participant is unable to take the survey due to cognitive impairment or another disability, they will identify an informant (e.g., a close family member, neighbor, or friend). The respondent will still be encouraged to participate in all sections of the study that they are able to; however, the informant will provide data when the participant is unable. Each respondent will be provided with a small monetary token of appreciation to compensate for their time.

The LOSHAK field team for the feasibility and pilot phase of the study consists of one overall study coordinator, four interviewers, one field coordinator/supervisor, and one quality assurance officer. All interviewers undergo two weeks of rigorous training that involves the following content: project introduction and summary; familiarization with the study area and population; approaching and consenting participants; computer-assistant personal interview (CAPI) training; introduction to survey measures; cognitive tests training; methods to perform physiological and anthropometric measurements; techniques for performing dried blood spot collection, drying, storage, and transportation of the DBS samples to a central facility in Nairobi; air pollution monitor handling and usage; respondent remuneration; and uploading data. The coordinator and supervisor have extensive experience in this role within the study area, including experience in performing large population-based surveys. In addition, the study team has a long-standing relationship with community health unit leaders, community health volunteers, and community leadership. All survey instruments and data collection protocols will undergo pretesting (and revisions as appropriate) prior to data collection to identify any problems with the survey instruments and data collection protocols, as well as to assess interviewer readiness.

### Consent

Consent is obtained for each sub-section of the study to gauge the acceptability of each study measure and protocol. Separate informed consent will be obtained for (i) survey questions; (ii) wearable air pollution monitoring; (iii) physiological and anthropometric measures; (iv) collection of dried blood spots. Consent will be obtained directly from respondents. In cases where they are unable to provide consent due to cognitive impairment or disability, consent will be obtained from a close informant, as previously described. In cases where a respondent is unable to read, the informed consent documents will be read out loud by the interviewer, and for those who cannot sign a thumb impression will be collected confirming their consent in the presence of a witness.

### Data Management and Analysis

Questionnaire data and sample information from households will be collected using the ODK platform (Get ODK Inc., San Diego, CA, USA) using Android tablets. Once data are uploaded to AKU’s online data repository, the data undergo real-time quality assurance and automated error reports are used to prompt corrective action in the field. Study participants are given unique identifiers and a separate log is maintained to protect the identities of study participants.

To support the objectives of this feasibility and pilot phase of LOSHAK, extreme data values will be identified, with careful attention to recognizing systematic reasons for skewness or extreme values. Of key importance at this stage is a full interrogation of missing data and the reasons for missingness (e.g., don’t know, refused, illiterate, etc.). We will also carefully examine consent rates for each section of the study. Next, to ensure analyses are guided by theory and hypothesis, we will conduct confirmatory factor analyses to test fits for models of hypothesized constructs (e.g., general cognitive performance, memory, depression, etc.). Adequate fit will be evidence that such domains can be used to reliably rank participants in this study with respect to constructs of interest (e.g., cognitive performance) and to classify impairments. In cases where we identify suboptimal fit, we will use empirical results and theory to guide the revision of the instrument and subsequent piloting of the revised version prior to the next phase of LOSHAK.

Dried blood spot assay values will be summarized, and we will compare results from the AKU and University of Washington laboratories, assessing for systematic differences. This analysis will be conducted in concert with the NIH-funded Biomarker Network. Data from mobile air pollution monitors will be downloaded, cleaned, and summarized. We will assess wear time, data capture, and plausibility of values.

### Ethical Considerations

The Institutional Scientific and Ethical Review Committee at Aga Khan University, Kenya and the Institutional Review Board at the University of Michigan have both reviewed and approved LOSHAK. Approval to undertake LOSHAK was also granted by appropriate national and local governmental bodies. The LOSHAK team regularly engages with local community representatives in Kilifi County and local governmental administrative officers, and community/stakeholder engagement will receive even greater emphasis in the next phase as LOSHAK expands to include communities from throughout Kenya. The LOSHAK team is committed to open access and data-sharing principles. Data will be made freely accessible in online digital repositories.

## Conclusion

This study has been designed to build the necessary infrastructure to collect data that will promote optimal health and economic well-being in Kenya, and more generally in SSA, in the setting of rapid population aging. Accordingly, LOSHAK presents a novel opportunity to elucidate key issues in aging in SSA, including primary risk factors for ADRD, mental health conditions, and disability; the role of environmental pollution and climate change; and the factors that influence late-life economic well-being. As a member of the family of HRS studies, data from LOSHAK (loshak.org) will be widely accessible and harmonized with other network studies to enable cross-national comparisons.
